# Antimicrobial susceptibilities and genomic epidemiology of *Neisseria gonorrhoeae* in Stockholm, Sweden

**DOI:** 10.1007/s10096-023-04633-6

**Published:** 2023-07-13

**Authors:** Nora Vestberg, Karin Haij Bhattarai, Hong Fang

**Affiliations:** Department of Clinical Microbiology, Karolinska University Hospital, Karolinska Institutet, SE-141 86 Stockholm, Sweden

**Keywords:** *Neisseria gonorrhoeae*, Gonococci, Antimicrobial resistance, Extended-spectrum cephalosporins, Cefixime, Ceftriaxone, High-level azithromycin resistance, Whole-genome sequencing, Multi-locus sequence type, NG-STAR

## Abstract

The aim of this study was to investigate the genomic epidemiology and antimicrobial susceptibilities of *N. gonorrhoeae* isolates in Stockholm, Sweden. In total, 6723 isolates detected in Stockholm, Sweden, from January 2016 to September 2022, were examined for antimicrobial susceptibilities by using *E*-test. Whole-genome sequencing (WGS) was applied to isolates in sentinel surveillance and isolates resistant to extended-spectrum cephalosporins (ESCs) or high-level azithromycin (HLAzi-R, MIC ≥ 256 mg/L). As sentinel surveillance, consecutive clinical isolates (*n* = 396) detected every 4th week from January 2021 to September 2022 were enrolled in the study. Of the 6723 isolates investigated, 33 isolates (< 1%) were found to be resistant to cefixime, one of which was co-resistant to ceftriaxone and ciprofloxacin and was detected in September 2022. Ten isolates presented a high level of azithromycin resistance. Resistant rates to ciprofloxacin varied from 32 in 2017 to 68–69% in 2021–2022. Elevated MIC_50_ and MIC_90_ of azithromycin were observed over the years. No resistance to spectinomycin was identified. The most frequently occurring MLST in the sentinel surveillance was ST9362 (23%), followed by ST11706 (9%), ST7359 (8%), ST10314 (7%), and ST11422 (6%). The ceftriaxone-resistant isolate belonged to ST8130 and the novel NG-STAR ST4859. Genomic resistance traits found in this strain included mutations in genes *mtrR* (A39T), *parC* (S87N), and *gyrA* (S91F and D95A), as well as the presence of *bla*TEM-135 and *tetM* genes. A predominance of ST9362 was observed in Stockholm. The high number of azithromycin and ciprofloxacin-resistant isolates and the emergence of a strain with a novel NG-STAR are of great concern.

## Introduction

Gonorrhea is a sexually transmitted infection (STI) caused by *Neisseria gonorrhoeae.* Every year, 78 million new cases are estimated, making it the second most common STI worldwide, next to chlamydial infection [1]. In Sweden, 3355 cases of gonorrhea were reported to the Public Health Agency in 2022, corresponding to 32.1 cases per 100,000 inhabitants [2]. An alarming increase in national gonorrhea incidence has been observed from 7.8 to 32.1 cases per 100,000 inhabitants in the years 2009–2022 [3]. Stockholm, the capital city of Sweden, has the highest gonorrhea incidence in the country. In 2022, 55.5% of all cases in Sweden were identified in Stockholm with an incidence of 77.1 cases per 100,000 inhabitants [2].

Antimicrobial-resistant *N. gonorrhoeae* is an emerging and significant public health concern. In 2020, about half of all detected strains were estimated to be resistant to at least one antibiotic used today [4]. *N. gonorrhoeae* was one of twelve species on the World Health Organization’s (WHO) priority list of pathogens in need of research and development of new antimicrobial drugs in 2017 [5]. It has developed resistance to almost all antimicrobial drugs previously used for the treatment of gonorrhea, such as penicillins, tetracyclines, and fluoroquinolones [1]. Antimicrobial resistance (AMR) mechanisms in gonococci include chromosomal mutations, resistance plasmid conjugations and external gene transfer [6]. Most AMR determinants are chromosomally situated except for the *bla*TEM genes and the *tetM* gene which encode β-lactamase production and tetracycline resistance, respectively [7]. Resistant *Neisseria* spp. may result from exposure to antimicrobials leading to spontaneous gene mutations and/or the acquisition of resistance genes. Commensal *Neisseria* spp., especially those colonizing the pharynx, might often be exposed to antimicrobials, making it a reservoir for AMR genes. Due to pharyngeal gonorrhea often being asymptomatic, *N. gonorrhoeae* and other *Neisseria* spp. can coexist and transfer resistance genes. *Neisseria meningitidis*, a common colonizer of the oropharynx, have also shown increased development of resistance to antimicrobial agents. Transformation or conjugation events or point mutations give rise to decreased antimicrobial susceptibility. However, alleles encoding resistance might have appeared before the antibiotic era and so have been selected in the use of antibiotics [8]. Reduced susceptibility for cephalosporins and quinolones has been observed in populations with high consumption of these antimicrobials [9]. In *N. gonorrhoeae*, horizontal gene transfer is likely involved in the emergence of mosaic *penA* alleles, developing in decreased susceptibility or resistance to extended-spectrum cephalosporins (ESCs) [6, 7]. The *penA* gene encodes altered forms of penicillin-binding protein 2 (PBP2) which is the target protein for β-lactam antibiotics. The mutations can be mosaic and non-mosaic. The mosaic *penA* mutation contains up to 60–70 amino acid alterations compared to the wild type [6, 7], while non-mosaic alleles have 1–13 mutations in the C-terminal [10]. Increased MICs of ESCs may also result from MtrCDE membrane pump protein overproduction, most commonly from a substitution (G45D) in the MtrR protein or a deletion of −35A in the *mtrR* promoter region. However, these mechanisms alone do not determinate resistance [10].

In Sweden, for uncomplicated gonorrhea, the first-line treatment is a single intramuscular dose of 1 g ceftriaxone [11]. Monotherapy with azithromycin is not recommended due to concerns about antimicrobial drug resistance development [10]. Isolates with high-level resistance to azithromycin (MIC ≥ 256 mg/L) have been identified in Sweden, England, Scotland, Argentina, Italy, and the USA, among other countries [7]. High-level azithromycin resistance is associated with an A2059G mutation in 23S rRNA [12]. Since 2009, a decrease in isolates resistant to ESCs has been observed in the EU/EEA. This may partly be an effect of the changed European treatment guidelines in 2012, in which cefixime 400 mg was replaced by ceftriaxone 500 mg plus azithromycin 2 g [3]. Although a decrease in resistance to ESCs has been observed in the EU/EEA and Sweden lately, concerns of future resistance remain, especially to ceftriaxone. Monitoring antimicrobial susceptibilities is a public health priority to address the emerging threat of antimicrobial-resistant gonorrhea.

The aim of this study was to investigate the genomic epidemiology and antimicrobial susceptibilities of *N. gonorrhoeae* isolates in Stockholm, Sweden.

## Material and methods

### Bacterial isolates

Antimicrobial susceptibility testing was performed on 6723 clinical isolates detected from January 2016 to September 2022 (one isolate per patient and infection episode).

Whole-genome sequencing (WGS) was applied to two groups of isolates: the epidemiological surveillance and isolates resistant to extended-spectrum cephalosporins (ESCs) or high-level azithromycin (HLAzi-R, MIC ≥ 256 mg/L). As sentinel surveillance, consecutive clinical isolates detected every 4th week from January 2021 to September 2022 (*n* = 396) were enrolled in the surveillance group. Genomic-resistant traits in 33 ESC-resistant isolates and ten HLAzi-R isolates were examined. All isolates were collected at the Department of Clinical Microbiology, Karolinska University Hospital, Stockholm, Sweden.

### Antimicrobial susceptibility testing

The minimum inhibitory concentrations (MICs) of azithromycin, cefixime, ceftriaxone, ciprofloxacin, and spectinomycin were determined by using gradient *E*-test (bioMérieux, France) on chocolate agar incubated for 24 h at 36 °C 6.0% CO_2_. Quality control was performed using the *N. gonorrhoeae* reference strains CCUG 41811 and CCUG 41812. EUCAST clinical breakpoints were used to categorize isolates as resistant or susceptible to all antibiotics except azithromycin [13]. No clinical breakpoints are published for azithromycin; the EUCAST epidemiological cut-off value (ECOFF) of 1 mg/L was used.

### Whole-genome sequencing

Genomic bacterial DNA was extracted using the EZ1 Advanced XL (QIAGEN) according to the manufacturer’s instructions. Sequencing was performed on the Illumina platform at SciLifeLab (Stockholm, Sweden), generating paired-end sequences with ≥ 30× coverage. Multi-locus sequence typing (MLST), core-genome MLST (cgMLST), N. gonorrhoeae sequence typing for antimicrobial resistance (NG-STAR), and genomic resistance traits were analyzed with the 1928 Diagnostics online platform (1928 Diagnostics, Gothenburg, Sweden). NG-STAR allelic profiles were examined using the NG-STAR version 2.0 website (NG-STAR Government of Canada) [14].

## Results

### Antimicrobial susceptibility

Of the 6723 clinical isolates tested, 33 isolates (< 1%) were resistant to cefixime with MIC-values ranging from 0.19 to 1 mg/L, one of which was also resistant to ceftriaxone and ciprofloxacin, detected in September 2022. Resistance rates to ciprofloxacin varied from 32 to 69% with the highest rates in 2022 (Jan–Sep) and 2021 (Table 1). MIC_50_ and MIC_90_ for ciprofloxacin and azithromycin, representing the MIC value at which growth was inhibited in 50% and 90% of isolates, respectively, are presented in Table 2. During the whole study period, ten high-level azithromycin-resistant isolates with MIC ≥ 256 mg/L were identified. No resistance to spectinomycin was observed.

**Table 1:** Antibiotic resistance rates (%) of clinical *N. gonorrhoeae* isolates collected at the Department of Clinical Microbiology, Karolinska University Hospital, Stockholm, Sweden, from January 2016 to September 2022

	2016	2017	2018	2019	2020	2021	2022 (Jan–Sep)
	n = 799	*n* = 1258	*n* = 1200	*n* = 1210	*n* = 975	*n* = 742	*n* = 539
Cefixime	1.7%	< 1%	< 1%	< 1%	< 1%	< 1%	< 1%
Ceftriaxone	0	0	0	0	0	0	< 1%
Ciprofloxacin	43%	32%	52%	57%	54%	69%	68%
Azithromycin^a^	2.4%	4.4%	9%	16%	23%	34%	28%
Spectinomycin	0	0	0	0	0	0	0

^a^Azithromycin MIC >1 mg/L

**Table 2:** MIC_50_ and MIC_90_ of ciprofloxacin and azithromycin. MIC_50_ is the MIC value at which growth was inhibited in 50% of isolates while MIC_90_ is the MIC value at which growth was inhibited in 90% of isolates

	2016	2017	2018	2019	2020	2021	2022
Ciprofloxacin MIC_50_	0.125	0.004	0.25	1	1	2	2
Ciprofloxacin MIC_90_	16	12	12	8	8	8	8
Azithromycin MIC_50_	0.25	0.25	0.25	0.38	0.5	1	0.5
Azithromycin MIC_90_	0.5	0.75	1	2	2	2	2

### Genomic epidemiology

**Fig. 1:**
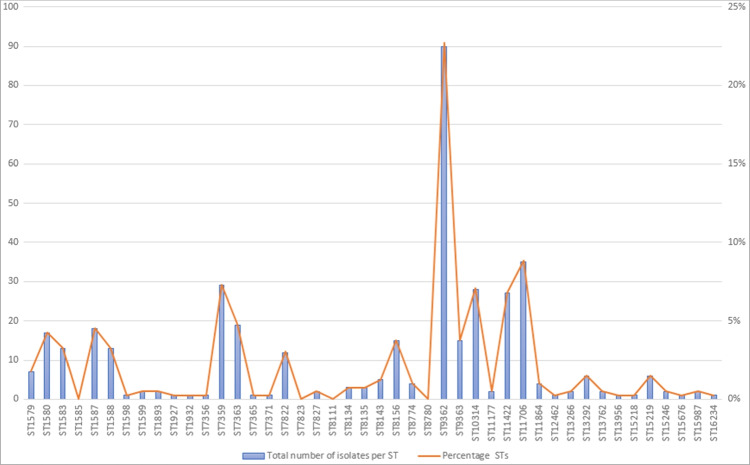
Prevalence of STs during the study period Jan 2021 to Sep 2022 in the total number of isolates per ST and percentage of each ST

Of the 396 *N. gonorrhoeae* isolates sequenced for epidemiological monitoring, 44 different STs were identified. The five most common STs during the whole study period of 22 months were ST9362, ST11706, ST7359, ST10314, and ST11422 (Fig. 1 and Table 3).

**Table 3:** Prevalence of the five most frequently occurring STs during the whole study period and distributed in years 2021 and 2022 (January–September)

	ST9362	ST11706	ST7359	ST10314	ST11422
2021	28%	10%	12%	7%	(2%)
2022 (Jan–Sep)	15%	7%	(1%)	8%	14%
2021–2022 (Jan–Sep)	23%	9%	8%	7%	6%

The four most frequently occurring STs during 2021 were ST9362, ST7359, ST11706, and ST10314. The four most frequently occurring ST during January to September 2022 was ST9362, ST11422, ST10314, and ST11706. ST9362 was the most dominant ST in both years. The prevalence of ST7359 decreased from 12 in 2021 to 1% in 2022; in the meantime, ST11422 increased from 2 to 14% (Table 3). When looking at the cgMLST of each of the five most prevalent STs, genetic clusters with an allelic difference of ≤ 5 were observed as follows: ST9362: ten clusters, ST11706: five clusters, ST7359: two clusters, ST10314: three clusters, and ST11422: two clusters.

Of the five most frequently occurring MLSTs during the study period, both ST9362 and ST10314 had a novel NG-STAR type as the dominating variant (80% and 85% respectively). The allelic profiles of the most dominant novel NG-STAR types are presented in Table 4. For ST11706, 74% of the isolates were NG-STAR ST1869. For ST7359, NG-STAR ST231 was dominating (97%) and for ST11422, most of the isolates belonged to NG-STAR ST193 (88%) (Table 4).

**Table 4:** Most common NG-STAR types among the five most frequently occurring MLSTs during the study period. For the novel NG-STAR types, the most occurring novel allelic profile is presented

MLST	NG-STAR	Novel allelic profile
		*penA, mtrR, porB, ponA, gyrA, parC*, 23S sRNA
ST9362	Novel (80%)	166, 1, 100, 100, 7, 3, 100
ST11706	1869 (74%)	
ST7359	231 (97%)	
ST10314	Novel (85%)	288, 39, 3, 1, 7, 3, 100
ST11422	193 (88%)	

### High-level azithromycin-resistant isolates

**Fig. 2:**
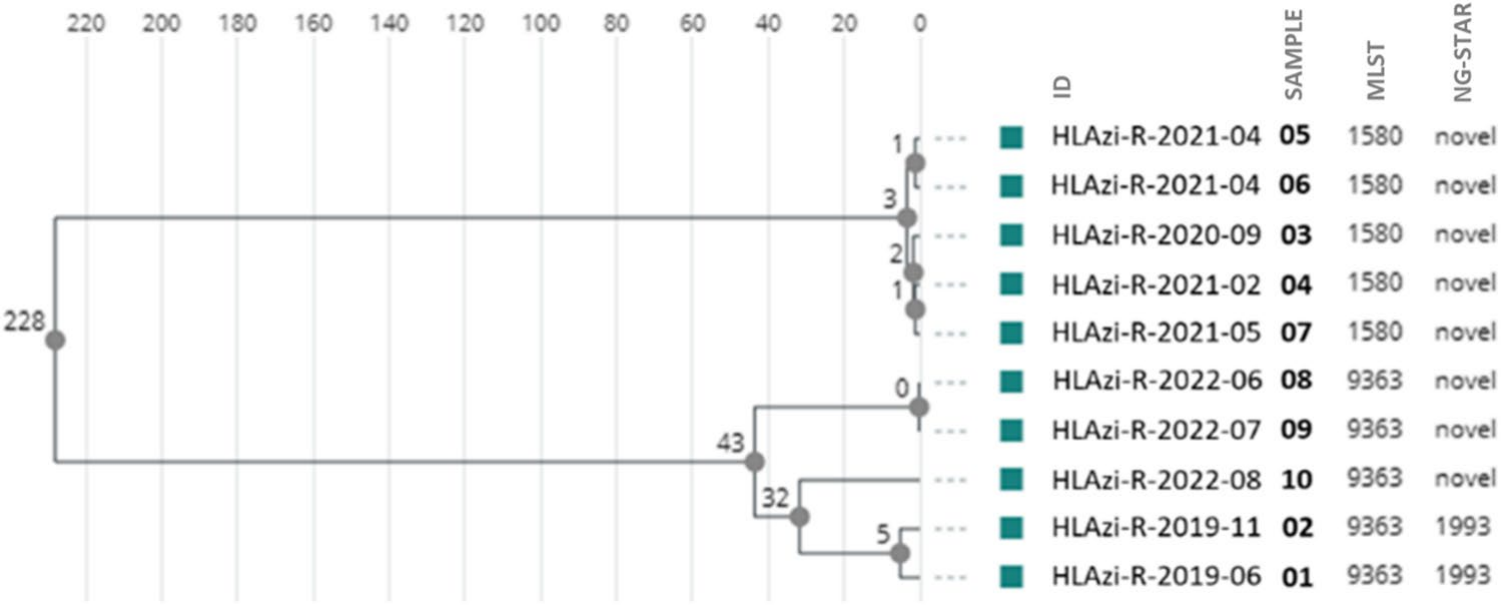
Phylogenetic tree of high-level azithromycin-resistant isolates using a cgMLST scheme. Three genetic clusters with an allelic difference of ≤ 5 were observed

Of the ten isolates with high-level azithromycin resistance, five belonged to ST1580 and five to ST9363, all carrying the 23S rRNA A2059G mutation. All five ST1580 isolates presented the same novel NG-STAR ST (*penA*1444, *mtrR*311, *porB*3, *ponA*100, *gyrA*100, *parC*100, and 23S1), while two different novel NG-STAR types and NG-STAR ST1993 were observed in the ST9363 isolates. The cgMLST phylogenetic tree generated three genetic-related clusters when a cut-off of ≤ 5 allelic differences were applied. The isolates in each cluster harbored the same MLST type and the same NG-STAR type (Fig. 2).

### ESC-resistant isolates

Of the 33 cefixime-resistant isolates, 29 were ST7363, two ST1579, one ST1901, and one ST8130. Of the ST7363 isolates, all but one carried a non-mosaic *penA* type XIV allele with A517G mutation (NG-STAR *penA* allele 14). Of all the cefixime-resistant isolates, 12 had the *mtrR*-35A deletion while fifteen had the *mtrR* G45D substitution. The predominating NG-STAR types were 232 (36%, *n* = 12) and 854 (27%, *n* = 9).

One isolate from September 2022 was simultaneously resistant to cefixime (MIC 1 mg/L), ceftriaxone (MIC 0.25 mg/L), and ciprofloxacin (MIC 8 mg/L). It belonged to ST8130 and the novel NG-STAR type ST4859. The isolate harbored *mtrR* A39T, *parC* S87N, and *gyrA* S91F and D95A mutations. Other identified resistance genes were *tetM* (tetracycline) and *bla*TEM-135 (broad-spectrum beta-lactamase). Further susceptibility testing of the isolate showed resistance to cefotaxime (MIC 1 mg/L). The MIC value for doxycycline was 24 mg/L, while there are no published S/R breakpoints for *N. gonorrhoeae* of doxycycline.

## Discussion

The increased incidence of reported gonorrhea cases worldwide combined with the rapid development of AMR is a critical global health concern. Decreased susceptibility globally to the last-line third-generation cephalosporin ceftriaxone is worrying. Therefore, thorough surveillance of phenotypic and genotypic AMR patterns is crucial.

An increased rate of isolates resistant to ciprofloxacin or azithromycin was observed during the study period. In 2017, 32% of the isolates were resistant to ciprofloxacin compared to 69% in 2021. MIC_50_ for ciprofloxacin increased from 0.125 in 2016 to 2 mg/L in 2022, while MIC_90_ decreased from 16 in 2016 to 8 mg/L in 2022. Ciprofloxacin has been the first-line treatment for many years. However, since the early to mid-2000s, many Asian and European countries have removed ciprofloxacin as a first-line treatment option. The increased resistant rates in Europe might be partly due to imported cases [7]. The percentage of azithromycin-resistant isolates (based on ECOFF) increased from 2.4 in 2016 to 34% in 2021. In the last 4 years (2019–2022), the MIC_90_ for azithromycin was 2 mg/L, higher than the epidemiological cut-off value (ECOFF) of 1 mg/L. A similar situation was described in a Swedish national report from 2021 (Swedres-Svarm 2021), remaining unknown if clinical treatment with azithromycin 2 g would be successful [11].

The first high-level azithromycin-resistant isolate in Sweden was found in 2011 [15]. During the whole study period, 10 isolates with an azithromycin MIC ≥ 256 mg/L were detected. WGS of the isolates resulted in five ST1580 and five ST9363, STs which have also been reported in Ireland and South America, among other countries [16, 17]. All the isolates carried the 23S rRNA A2059G mutation, which was supposed to be associated with high-level azithromycin resistance [16]. Recently, it has been reported that azithromycin resistance could also be driven by mosaic sequences in the *mtrR* promoter and *mtrD* gene of the MtrRCDE efflux pump system [17]. However, the MtrRCDE efflux pump system was not further investigated in the present study.

In a 2016 national study of clinical isolates in Sweden, the five most common STs were ST8156, ST7363, ST1901, ST1588, and ST7359 [3]. All these STs were represented in the present epidemiological monitoring study except for ST1901. However, a ST1901 strain showed up in December 2022 in our continued sentinel surveillance. In this study, ST9362, ST11706, and ST10314 were among the five most common STs both in 2021 and 2022 (Jan–Sep). Notably, ST7359 decreased from 12 in 2021 to 1% in 2022 (Jan–Sep), while ST11422 increased from 2 in 2021 to 14% in 2022 (Jan–Sep). This indicates the dynamic presence and spreading of certain strains in Stockholm. Among the five most common STs in the present study (ST9362, ST11706, ST7359, ST10314, and ST11422), several genetic clusters were identified by cgMLST analysis, indicating possible clonal relationship within the cluster.

ST7363 was the predominant sequence type (88%) among the isolates resistant to ESCs in Stockholm during the study period. Among the cefixime-resistant ST7363 isolates, the most common NG-STAR types were 232 (36%) and 854 (27%). The non-mosaic *penA* type XIV allele with the A517G mutation has been reported to be associated with decreased ESC susceptibility [18]. In the present study, this resistance mechanism was identified in 85% of cefixime-resistant isolates.

Although not listed as top five STs during the study period (2021–2022), ST7363 was the sixth common ST with a prevalence of 4.8% in Stockholm. In 2013, it was one of the most prevalent STs in 20 different EU/EEA countries [19]. In that study, only a small cluster was cefixime-resistant, and those isolates were phylogenetically unrelated to the main cluster [19]. Besides ST7363, two ST1579 and one ST1901 isolates were found to be resistant to cefixime in the present study, which was in accordance with reports of ST1901 and ST1579 being spread worldwide and associated with cefixime resistance [20, 21]. No cefixime-resistant isolates were simultaneously resistant to azithromycin in the present study.

The recently detected cefixime-resistant isolate also presented resistance to ceftriaxone and ciprofloxacin. It belonged to ST8130 and the novel NG-STAR ST4859. The isolate harbored *mtrR* A39T, *parC* S87N, and *gyrA* S91F and D95A mutations. The *mtrR* A39T mutation has been reported to be associated with the multi-drug efflux pump. The *parC* and *gyrA* mutations were responsible for fluoroquinolone (ciprofloxacin) resistance [22]. Other resistance genes identified were *tetM* (tetracycline) and *bla*TEM-135 (broad-spectrum beta-lactamase). Tetracycline was not available at the laboratory, but the isolate showed a MIC of 24 mg/L for doxycycline (clinical breakpoints not available [13]). Tetracycline-resistant gonococci were widespread internationally [7]. The *bla*TEM-135 genes have been reported to be located on β-lactamase plasmids, and the most common ones were the Asian, African, and Toronto/Rio plasmids [23]. Isolates carrying the *bla*TEM-135 genes have been found in China, Argentina, and Poland, among other countries [23-25]. Sweden was one of the countries having the highest percentage of beta-lactamase-producing *N. gonorrhoeae* in Europe in 2016 [26]. It is much concerned that the extensive use of ESC for gonorrhea treatment creates the possibility for the selection of new variants of TEM beta-lactamase, including extended-spectrum beta-lactamases (ESBLs).

Since 2019, ceftriaxone 1 g monotherapy has been the Swedish guideline recommended for uncomplicated gonorrhea due to the low prevalence of ceftriaxone resistance [6]. Since ceftriaxone is the last-line alternative for monotherapy, the emergence of a novel ceftriaxone-resistant isolate in Sweden is worrying. Dual therapy treatment with ceftriaxone and azithromycin was earlier recommended in Sweden and is being used in many countries worldwide. However, the rapid expansion of isolates with decreased susceptibility to azithromycin and the presence of high-level resistant isolates are of great concern. Although no resistance to spectinomycin has been observed, the drug is not a suitable treatment option due to its not being available in many countries, the lack of effective treatment of pharyngeal gonorrhea, and the observed rapid development of resistance when being used as first-line therapy in Korea in the 1980s [6].

The number of isolated strains decreased during the COVID-19 pandemic (2020–2021) compared to previous years. This decrease could possibly be a result of social and physical restrictions and a potential reduction in healthcare access for testing and treatment due to the pandemic. Interestingly, the level of isolates resistant to ciprofloxacin and azithromycin was at the highest level of the study period in 2021. A study from the Netherlands observed a shift during the pandemic from ST8156 being the most common ST before lockdown (Jan–Feb of 2020) to ST9362 being the most frequently occurring ST during lockdown (May–June of 2020) [27]. Similar to the finding in Amsterdam, ST9362 predominated (28%) in Stockholm, Sweden, in 2021, when the second wave of COVID-19 hit. The percentage of ST9362 decreased in the first 9 months of 2022 to almost the same level as the runner-up ST11422 (15% and 14% respectively). However, since we have no sequencing data before 2021, no further conclusions can be drawn.

The emergence and increase of AMR in *N. gonorrhoeae* need to be delayed by a thorough and rapid detection of AMR variants combined with extensive transmission-preventative actions. Furthermore, novel antimicrobials or other therapeutic medical products need to be developed for effective monotherapy.

## Conclusion

Incidences of *N. gonorrhoeae* isolates resistant to ciprofloxacin or azithromycin have been increasing in Stockholm, Sweden, between the years 2016 and 2022. The emergence of ceftriaxone-resistant isolates in Sweden and other countries worldwide is of great concern. ST9362 was the most common sequence type in Stockholm in 2021–2022, although a dynamic prevalence was observed. Multiple actions are required to address the problem and to avoid untreatable gonorrhea in the future.

## Data Availability

The datasets generated and analyzed during the current study are not publicly available but are available from the corresponding author upon a reasonable request.
